# Fabrication of Naturally Derived Chitosan and Ilmenite Sand-Based TiO_2_/Fe_2_O_3_/Fe-N-Doped Graphitic Carbon Composite for Photocatalytic Degradation of Methylene Blue under Sunlight

**DOI:** 10.3390/molecules28073154

**Published:** 2023-04-01

**Authors:** Amavin Mendis, Charitha Thambiliyagodage, Geethma Ekanayake, Heshan Liyanaarachchi, Madara Jayanetti, Saravanamuthu Vigneswaran

**Affiliations:** 1 Faculty of Humanities and Sciences, Sri Lanka Institute of Information Technology, Malabe 10115, Sri Lanka; 2 Faculty of Engineering and Information Technology, University of Technology Sydney, P.O. Box 123, Sydney, NSW 2007, Australia; 3 Faculty of Sciences & Technology (RealTek), Norwegian University of Life Sciences, P.O. Box N-1432 Ås, Norway

**Keywords:** chitosan, ilmenite, graphitic carbon, photocatalysis, sunlight

## Abstract

Fabrication of chitosan and ilmenite sand-based novel photocatalysts through the catalytic graphitization of chitosan is reported. Nanocomposites consisted of TiO_2_, Fe_2_O_3_ and Fe nanoparticles dispersed on a nitrogen-doped graphitic carbon framework. The surface area, pore volume and macropore structure of the carbon matrix is disturbed by the heterogeneously distributed nanoparticles. The extent of graphitization expanded with increasing metal loading as indicated by variation in the *I_D_*/*I_G_* ratio. The nanomaterial’s surface consists of Fe^3+^ and Ti^4+^, and graphitic, pyridinic and pyrrolic nitrogen were found in the carbon matrix. The band gap values of the composites varied in the 2.06–2.26 eV range. The photocatalytic activity of the synthesized nanomaterials was determined, and the highest rate constant for the photodegradation of methylene blue under sunlight was 4.4 × 10^−3^ min^−1^, which resulted with 10 mg/L MB and 25 mg of the best-performing catalyst. The rate constant rose with increasing concentrations of persulfate added to the medium. The rate constant greatly diminished with the addition of isopropyl alcohol as it scavenged hydroxyl radicals. The presence of co-pollutants including Pb^2+^, rhodamine B, PO_4_^3−^ and Cl^−^ curtailed the rate of reaction. The activity reduced with an increasing number of uses of the catalyst.

## 1. Introduction

Chitin and its deacetylated derivative, chitosan, are a family of linear polysaccharides consisting of variable amounts of N-acetyl-2 amino-2-deoxy-D-glucose (glucosamine, GlcN) and 2-amino-2-deoxy-D-glucose (N-acetyl-glucosamine, GlcNAc) residues. Chitin is the most prevalent biopolymer in nature and a precursor of chitosan; it is present in a variety of eukaryotic species including crustacea, insects, and fungi [1]. Chitin or poly (β-(1→4)-N-acetyl-D-glucosamine) is a natural polysaccharide of major importance that was first identified in 1884 [2]. Organisms exhibit variable chitin content (wt%): crustacean shell waste typically contains 30% to 50% by weight of calcium carbonate and 20% to 30% by weight of chitin, whereas some genera of lobsters, including *Nephrops* sp. and *Homarus* sp., have the largest amount of chitin of any species that contain chitin [1]. N-acetyl-D-glucosamine is a building block of chitin, a polymer that is known as chitosan when it has undergone deacetylation and the repetitive units are mostly free of the acetyl functional group [2,3,4]. The mole fraction of the N-acetylated repeating units is defined as the degree of acetylation (DA), while the percentage of the repeating units of β-1,4-D-glucosamine in the polysaccharides is defined as the degree of deacetylation (DD) [2,5]. Along with molecular weight, degree of deacetylation is known to be a significant factor affecting the functions of chitosan.

The most frequently mentioned sources in the literature for the raw materials used to prepare chitosan are shrimp and crabs, although other species such as lobster, crayfish, and oyster have also been utilized [1]. Crab and shrimp shells are currently the primary commercial sources of chitin. In commercial processing, calcium carbonate is first dissolved by acid treatment to extract chitin, then proteins are dissolved using an alkaline solution. In order to get pure chitin that is colorless, a decolorization step is often used. However, due to variations in the ultrastructure of the initial material, all of those treatments must be tailored to the chitin source. Here, the purpose is to first yield high-quality chitin and then chitosan, which is obtained after partial deacetylation. However, chitin remains scarcely soluble when it is changed into various conformations [2].

Chitosan solubility depends on different factors such as polymer molecular weight, degree of acetylation, pH, temperature and polymer crystallinity. Homogeneous deacetylation (alkali treatment and low temperature) of chitin permits the production of polymers soluble in aqueous acetic acid solutions with DD as low as 28%, with this value never being reached under heterogeneous deacetylation (alkali treatment and high temperatures) [6]. Moreover, with a DD of 49%, the samples are soluble in water. This behavior is explained by the fact that homogeneous deacetylation first leads to an increase in the number of glucosamine units and then leads to a modification in the crystalline structure of the polymer [7].

Due to its distinctive biological and physiological properties such as solubility, biodegradability, biocompatibility, low reactivity and low allergencity, chitosan is well known for its wide range of applications, including medicine, pharmaceuticals, agriculture, food, cosmetics, the paper and textile industries and industrial sustainability [8,9,10,11]. Chitosan helps with the removal of dyes, metal ion binding, removal of pesticides and pharmaceuticals in water bodies, drug delivery, bio-imaging, wound dressing, advances in dentistry and ophthalmology, developing new nutraceuticals, tissue engineering, fiber-based scaffolds, controlled-release fertilizers, increasing water availability in plants, preventing abiotic stress in flora, antifungal activity [12,13], extending shelf life, new food packaging, developing new additives, emulsifiers and flocculants, developing novel hair styling gels, shampoos and hair serums. These are just some of the many applications of chitosan in the contemporary world [14].

Water scarcity is a major problem that affects every living being on earth and threatens their capacity to exist. Rapid and widespread industrialization releases a variety of dangerous substances, including but not limited to heavy metals, pesticides, fertilizers, medicines and dyes. Among them, dyes discharged into normal water reservoirs by the paper, paint, textile, cosmetics and plastic sectors constitute a major problem [15,16]. Due to their complex and synthetic composition, dyes are non-biodegradable and accumulate in water bodies, where they have severe adverse consequences. These potentially hazardous outcomes include reduced light penetration into water bodies (which limits photosynthesis), changes in biological and chemical oxygen demand and the accumulation of toxic materials such as heavy metals, surfactants, salts, chlorinated compounds etc. Additionally, the aesthetic worth is impacted by the dyes’ presence. Consequently, it is crucial to remove dyes from wastewater using a variety of techniques [17]. Many techniques have been deployed to remove dyes from wastewater including adsorption [18], membrane filtration [19], nanofiltration [20], coagulation and flocculation [21], oxidation [22], catalytic reduction [23] etc.

Ilmenite is a naturally available mineral found in igneous rocks, sediments and sedimentary rocks in many countries including Australia, South Africa, India, Brazil, Norway, Ukraine, Sri Lanka etc. Ilmenite’s composition varies depending on the location, but in general, XRF analysis has shown that it is composed of TiO_2_, FeO, Fe_2_O_3_, Na_2_O, SiO_2_, Al_2_O_3_, ZrO_2_, Cr_2_O_3_, MnO, CaO, MgO, SnO_2_, K_2_O, V_2_O_5_, P_2_O_5_ and SO_3_ [24]. To create pure TiO_2_ or to separate its component elements (Ti and Fe) for use in several applications, ilmenite is subjected to a variety of treatment conditions, most notably digestion in acids such as hydrochloric and sulfuric acids. TiO_2_ is well known throughout the world for its versatility in a variety of applications, such as photocatalysis to generate H2 [25,26,27] and the degradation of organic pollutants such as textile dyes [28,29]. The global textile industry contributes to economic growth, with China being the largest exporter of all fabrics, followed by the European Union, India and the United States [30]. However, one of the challenges with textile companies is the unacceptably toxic effluent, particularly with dyes, which are hard to degrade [31].

An essential procedure in the production of textiles is dyeing. The fibers are colored during this stage, and various chemicals may be added to facilitate the process of color adsorption onto the fibers. Some of these dyes and chemicals eventually wind up in the effluents created by the textile industry once the product is finished [32]. In addition to their unappealing appearance and toxic effects after breakdown, these dyes and chemicals may contaminate the surrounding soil, sediment and surface water, posing a serious threat to global environmental pollution [31]. Wastewater released from the dye industry comprises heavy metals, surfactants, chlorinated compounds, salts etc. in addition to the dyes which could severely impact aquatic life [33]. It is essential to treat textile effluents in order to safeguard the environment and make it possible to reuse the treated effluent in textile factory operations or for irrigation [31]. 

Dyes have been removed from wastewater by many techniques including adsorption [34], membrane filtration [35], coagulation and flocculation [36], oxidation [37] etc. Photocatalysis under advanced oxidation processes is a promising technique compared to the other methods as it completely degrades the dye molecules to harmless products. Photodegradation of methylene blue has been achieved under sunlight in the presence of different photocatalysts including TiO_2_/Seashell [38], TiO_2_@SiO_2_ [39], graphene oxide/WO_3_ [40], ZnO [41] and iron titanate [42], which are synthesized by chemical precursors. In this study, we report the synthesis of a novel nanocomposite using natural materials rather than expensive chemicals.

Chitosan from shrimp shells has good potential as a low-cost and environmentally friendly material, but currently, is it disposed of as a waste material. Ilmenite is a naturally available mineral which is rich in iron and titanium. In this study, we report the coupling of the derivatives of these two raw materials to fabricate a novel nanocomposite: one that is effective in removing dyes via both adsorption and photocatalysis. According to our knowledge, fabrication of photocatalysts using shrimp shells and natural ilmenite has not been reported elsewhere. We tried to add value to a waste product and a natural material in this study. Shrimp shells act as a nitrogen-rich carbon source, and ilmenite is the source of iron and titanium which catalytically graphitizes the carbon and serves as source for the semiconductors that generate charge carriers upon exposure to light. The conductive graphitic carbon matrix enhances the charge migration and hence facilitates the charge separation, which is essential for a high photocatalytic activity. Such eco-friendly nanocomposites were found to be promising candidates for environmental remediation in this study.

## 2. Results and Discussion

**Scheme 1:**
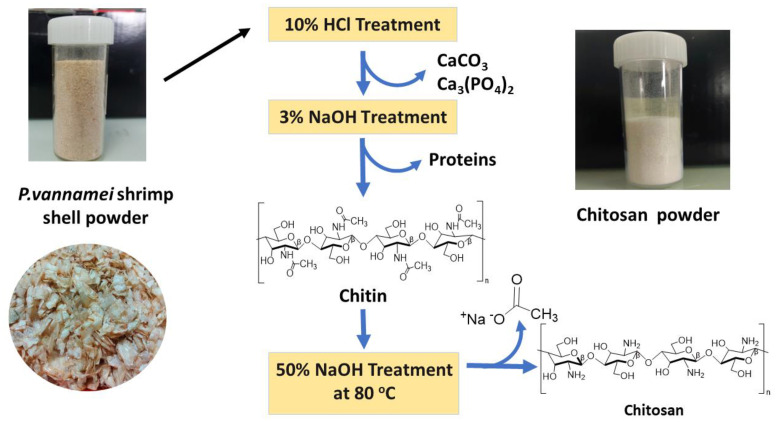
Production of chitosan from shrimp shells.

Shrimp shells were washed with distilled water and isopropyl alcohol to remove any impurities, and the dried shells were ground to produce a powder to increase the surface area, thus facilitating the treatments with chemicals in the subsequent steps. The obtained powder was treated with hydrochloric acid to remove CaCO_3_ and other minerals followed by treating the neutralized product with 3% NaOH to remove proteins. The deacetylation of the amide group at the second carbon of chitin was then achieved by treating with 50% NaOH heating at 80 °C. The detailed mechanism of producing chitosan from shrimp shells is shown in Scheme 1.

### 2.1. XRD Analysis

**Figure 1:**
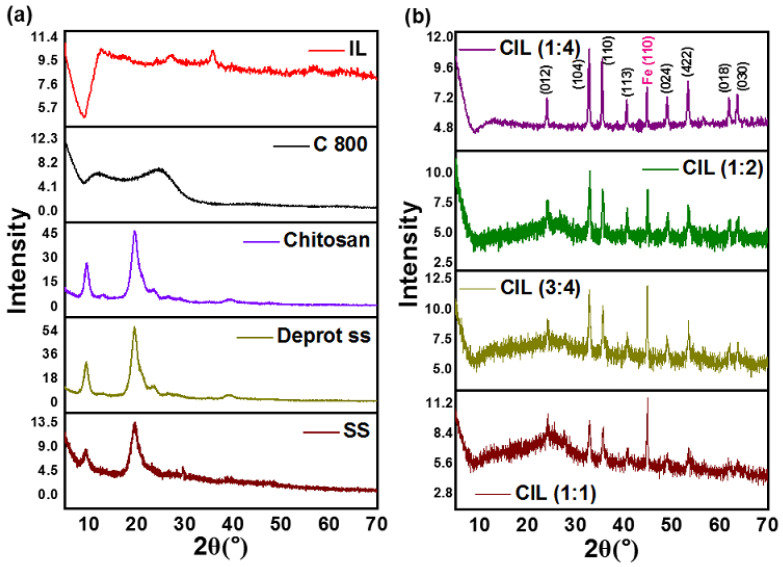
XRD patterns of the (**a**) raw materials and (**b**) fabricated nanocomposites.

The XRD patterns of the synthesized nanocomposites were collected to study the crystallographic orientation (Figure 1). The XRD pattern of the shrimp shells showed two significant peaks at 9.41° and 19.41° which are attributed to the (020) and (110) crystalline planes, while the small peak at 29.48° corresponds to the (104) crystalline peak of calcite. The XRD patterns of the deproteinated shrimp shells and the prepared chitosan show a shoulder peak at 20.50°, which is ascribed to the (120) crystalline peak, and the small broad peaks at 23.54°and 26.52° correspond to the (101) and (130) crystalline planes, respectively, in addition to the main peaks discussed above. The peak corresponding to calcite was not present in either deproteinated shrimp shells or chitosan, suggesting that all calcite was removed by the acid treatment (Supplementary Figure S1). The XRD pattern of the pyrolyzed chitosan exhibits a broad peak centered at 24.52° indicating the presence of amorphous carbon. The carbon–ilmenite composites reveal the peaks at 24.11°, 32.81°, 35.56°, 40.62°, 49.0°, 53.35°, 60.62° and 62.96°, which are attributed to (012), (104), (110), (113), (024), (422), (018) and (030) atomic planes of α-Fe_2_O_3_ (JCPDS card no: 41-1432), respectively. The d spacing values calculated by the Bragg’s law using Equation (1) are 0.3687, 0.2726, 0.2522, 0.2218, 0.2016, 0.1715, 0.1526 and 0.1474 nm, respectively. The position of the peak ascribed to the (101) plane did not significantly change in any composites, although a slight reduction was observed in CIL (1:4). The peak at 44.90° corresponds to the (110) plane of Fe(0) with a d spacing of 0.25 nm. The crystallite size was calculated by the Scherrer equation as shown in Equation (2), using the highest intense peak corresponding to the (110) plane for α-Fe_2_O_3_ and (110) for Fe(0). What emerged was that although the crystallite size of α-Fe_2_O_3_ was quite similar (around 39.65 nm) in all three CIL, i.e., (1:1), (3:4) and (1:2), it was significantly increased in the composite in which the chitosan was mixed with the ilmenite product at the ratio of 1:4 (55.39 nm). Similarly, the number of planes in the crystallites significantly increased to 202.67 in CIL (1:4) compared to the other composites. The crystallite size of Fe(0) in CIL (1:1) and CIL (3:4) were 60.7 and 61.9 nm, and the size in CIL (1:2) and CIL (1:4) was 89.8 nm. This indicates growth of the particle size with increasing metal oxide loading. Peaks corresponding to chitosan were not present in the composite since the structure of chitosan is converted to crystalline carbon by catalytic graphitization. However, the peak corresponding to the crystalline carbon (002) was not apparent in the XRD patterns of the composites because the concentration of the graphitic carbon made by the catalytic graphitization was not sufficient. All the parameters corresponding to the crystalline structures are tabulated in Table 1.

λ = 2dsin(θ)(1)

L = Kλ/β Cosθ(2)

where, λ—wavelength of the X-ray source; θ—diffraction angle; L—crystallite size; β—half maximum of the peak in radians; K—Scherer’s constant (0.9).

### 2.2. TEM Analysis

**Figure 2:**
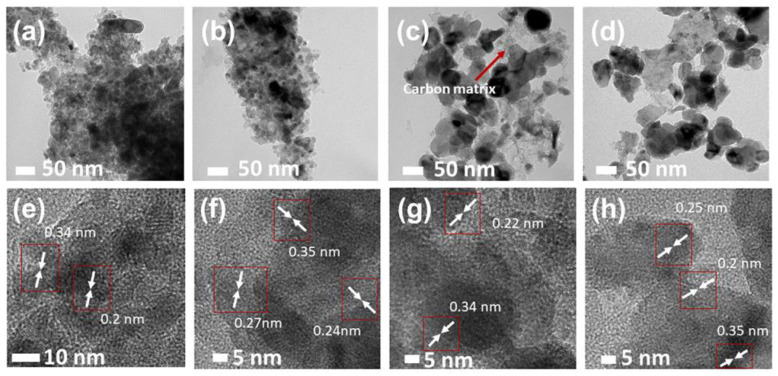
TEM images of (**a**) CIL (1:1), (**b**) CIL (3:4), (**c**) CIL (1:2) and (**d**) CIL (1:4). HRTEM images of (**e**) CIL (1:1), (**f**) CIL (3:4), (**g**) CIL (1:2) and (**h**) CIL (1:4).

TEM images were collected to study the morphology of the synthesized nanocomposites in the nanoscale context. Bright field TEM and HRTEM images of CIL (1:1), CIL (3:4), CIL (1:2) and CIL (1:4) are depicted in Figure 2. It could be seen that mostly spherical-shaped metal oxide nanoparticles along with some irregularly shaped nanoparticles were distributed on a nitrogen-rich carbon matrix of the TEM images of CIL (1:1) and CIL (1:2). However, agglomerated large nanoparticles are present in CIL (1:3) and CIL (1:4), indicating the growth of nanoparticles when increasing the proportion of the metal oxide product from ilmenite sand during the fabrication. The sizeof the smaller nanoparticles produced on the carbon matrix during the low temperatures of pyrolysis was increased due to Oswald ripening when the pyrolysis temperature was increased to 800 °C and maintained for 2 h. Small nanoparticles fuse with adjacent nanoparticles once the required energy is supplied during annealing to minimize the surface energy. It is apparent that the nanoparticles are heterogeneously distributed on the carbon framework. Nanoparticles were anchored to the nitrogen-containing functional groups of chitosan during hydrothermal treatment. However, during pyrolysis, different types of metal oxide nanoparticles fused with each other to form heterojunctions, and they migrated on the carbon matrix and further formed agglomerates, leading to heterogeneous distribution.

The HRTEM image of CIL (1:1) (Figure 2e) illustrates the lattice fringes with an interlayer distance of 0.25 nm and 0.35 nm, corresponding to the d spacings of (110) of α-Fe_2_O_3_ and (101) of TiO_2_ (Anatase). Meanwhile, the location of the lattice fringes of both compounds being adjacent to each other indicates the formation of heterojunctions of α-Fe_2_O_3_ and TiO_2_. The HRTEM image of CIL (1:2) (Figure 2f) shows the (104) plane of α-Fe_2_O_3_ represented by an interlayer distance of 0.27 nm in addition to the (110) atomic plane of α-Fe_2_O_3_. The d spacing of 0.22 nm shown in the HRTEM image of CIL (1:3) (Figure 2g) corresponds to the (113) atomic plane of α-Fe_2_O_3_, while the presence of (104) and (113) planes are indicated by the interlayer distances of 0.27 and 0.22 nm, respectively. The HRTEM image of CIL (1:4) (Figure 2h) shows the d spacings of 0.25, 0.20 and 0.35 nm corresponding to the (110) of α-Fe_2_O_3_, (110) of Fe(0) and (101) of TiO_2_ (Anatase), respectively. The calculated interlayer distance values are consistent with the d spacings calculated by the XRD data.

### 2.3. SEM Analysis

**Figure 3:**
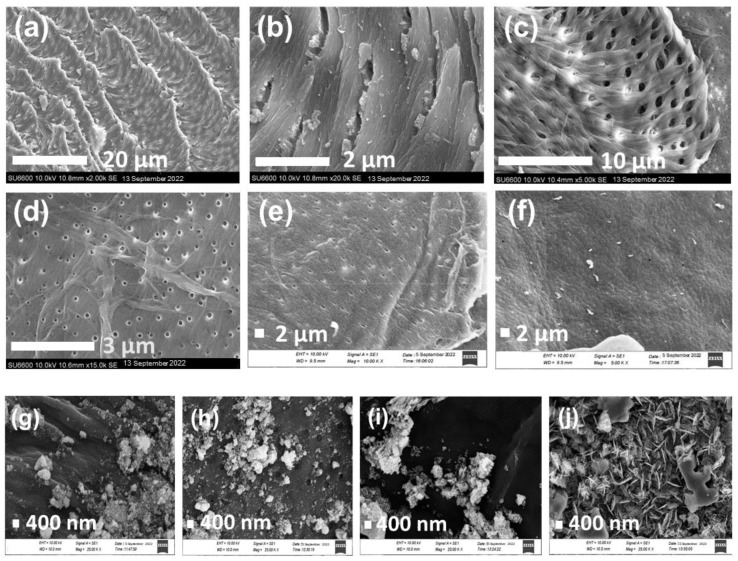
SEM images of (**a**) shrimp shells, (**b**) demineralized shrimp shells, (**c**) deproteinated shrimp shells, (**d**) chitosan, (**e**) pyrolyzed chitosan, (**f**) IL, (**g**) CIL (1:1), (**h**) CIL (3:4), (**i**) CIL (1:2) and (**j**) CIL (1:4).

SEM images of shrimp shells (Figure 3) showed a wave-like structure with inbuilt grooves, while SEM images taken at a high magnification, as displayed in Figure 3a, exhibited the detailed fine structure of the grooves and abundant small grooves. The shiny spots indicate the presence of CaCO_3_ and other metal-related compounds. An SEM image of demineralized shrimp shells is given in Figure 3b, and it shows a thread-like structure with clear circular holes. The mineralization partially hydrolyzes the polymeric structure where a fibrous network is more prominent, with holes indicating the removal of CaCO_3_ during the acid treatment. The SEM image acquired at higher magnification highlights the presence of voids and macropores among the fibrous structure. The SEM image of the deproteinated shrimp shells (Figure 3c) shows the presence of holes, but the well-defined fibrous network which was present in the demineralized shrimp shells was not present, and instead, the holes were present in a flat monotonous plane with few fibers. This suggests that the fibrous structure observed was the proteins available as a network on the shrimp shells.

The SEM image of the chitosan (Figure 3d) reveals the disappearance of most of the holes or macropores, and the surface of the pyrolyzed carbon is more homogeneous with minimum surface features. EDX analysis also further supported the observations made above. EDX analysis of the shrimp shells indicated the presence of Ca, Mg, P and O, which suggests the presence of the oxides and phosphates of Ca and Mg. These metal ions and phosphorous are not present in chitosan, indicating their removal via chemical treatment. The SEM image of the pyrolyzed chitosan (Figure 3e) shows the disappearance of porous structure. The SEM image of the metal oxides product, α-Fe_2_O_3_/TiO_2_, obtained from ilmenite (IL, Figure 3f) as described in the procedure, shows the presence of aggregated spherical nanoparticles. When they were hydrothermally coupled to chitosan followed by pyrolyzing at 800 °C as in CIL (1:1), (3:4), (1:2) and (1:4), it is more apparent that the aggregated nanoparticles were immobilized on the carbon matrix as shown in SEM images (Figure 3g–j, respectively). Interestingly, a different nanostructure was observed in the SEM image of CIL (1:4), where porous nanoplates were aligned vertically. Additionally, a differently shaped nanostructure formed due to the growth of nanomaterial during hydrothermal treatment was also present randomly on the carbon matrix (Figure 3j).

### 2.4. Resonant Raman Spectroscopy

**Figure 4:**
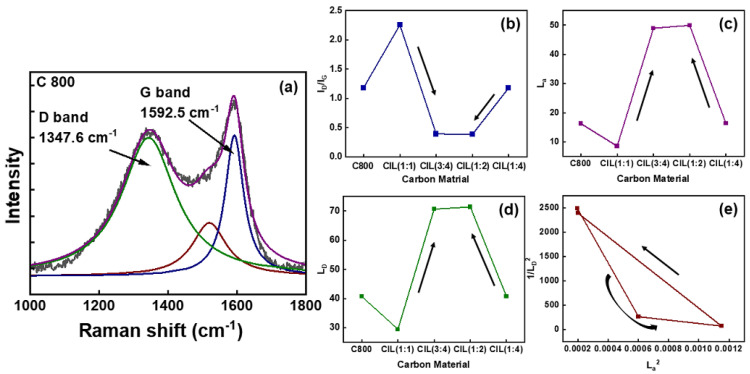
(**a**) Raman spectrum of C800 showing the peak deconvolution and variation of (**b**) *I_D_*/*I_G_*, (**c**) *L_a_*, (**d**) *L_D_* and (**e**) 1/*L_D_*^2^ vs. *L_a_*^2^.

Resonant Raman spectroscopy is a major characterization technique used to characterize carbon-based materials including amorphous carbon [43], carbon nanotubes [44], graphene [45], graphite [46] etc. The Raman spectrum of pyrolyzed chitosan is shown in Figure 4a. The peak at 1350 cm^−1^ is attributed to the D band and the peak at 1580 cm^−1^ is ascribed to the G band. The D band arises due to the second-order Raman process around the high symmetry κ-point involving defects and phonons of the A_1g_ symmetry. Furthermore, the D band represents the breathing modes of sp^2^ atoms in hexagons [47,48,49]. The G band is a first-order Raman process which occurs due to the doubly degenerate longitudinal optical (Lo) phonon mode occurring at the high symmetry Γ point [47,50]. Moreover, the G band represents the bond stretching of all pairs of sp^2^ atoms in both rings and chains [48,51]. The third peak at 1519.8 cm^−1^ appearing between the D and G bands represents the sp^2^ and sp^3^ carbons that are not being crystallized; it also occurs due to the hydrogen and oxygen in carbon [47].

The main parameter used to determine the degree of graphitization is the *I_D_*/*I_G_* ratio, where the intensity (*I*) represents the molecular vibrations entailed in the Raman process. There are two types of defects that could occur in two-dimensional lattices: point defects, which are also called 0D defects, and one-dimensional defects (1D). The distance between the nearest defects (*L_D_*) or the defect density is used to characterize the point defects where the *L_D_* reaches zero in highly disordered graphene while it is equal to infinity in pristine graphene. ID defects, which are characterized by the crystallite size (*L_a_*) and the crystallite area (*L_a_*^2^), are abundant in turbostratic carbon, whose structure is similar to graphite [52]. However, the planes could be rotated or translated to each other in a way that makes it different from graphite. *L_a_* and *L_D_* are calculated by the following equations [52,53,54].

(3) IDIG=102∓2LD2

(4) $$L_{a}\left(nm\right)=\left(2.14\times 10^{-10}\right)\times \lambda_{laser}^{4}\times {\left(\frac{I_{D}}{I_{G}}\right)}^{-1}$$

The sp^2^ to sp^3^ ratio of carbon materials determines the categorization of carbon in between amorphous carbon and graphite, where amorphous carbon with the highest sp^3^ content is called tetrahedral amorphous carbon (ta-C), and the sp^3^ content in graphite is zero. The amorphization trajectory is divided into three main transformations: graphite to nanocrystalline graphite (nc-G); nc-G to amorphous carbon (a-c); and a-c to ta-C. Graphite and nc-G contain 0% sp^3^ carbon, and a-c contains about 20% sp^3^ carbon [47]. The *I_D_*/*I_G_* ratio drastically increased with the introduction of the metal oxide product to chitosan in CIL (1:1), while the intensity ratio decreased with further increments of the proportion of the metal oxide incorporated, indicating the formation of nc-G, which is more ordered than the amorphous carbon (Figure 4b). The intensity ratio increased when the proportion increased from 66.67% (CIL (1:2)) to 80% (CIL (1:4)), indicating that nc-G becomes more graphitic. Consequently, it is suggested that the amorphous carbon material becomes more organized and forms nc-G when increasing the proportion of metal oxide to 66.67% from 50%. With a further increment of the metal oxide percentage from 66.7% to 80%, more graphitic carbon was formed. Both variations in *L_a_* and *L_D_* (Figure 4c,d) were consistent with the variation in the *I_D_*/*I_G_* ratio, indicating the formation of more ordered carbon. Moreover, variation in the density of 0D defects (1/*L_D_*^2^) as a function of the crystallite area (*L_a_*^2^) (Figure 4e) showed that the density of the 0D defects declines with increasing crystallite area, which further supports the observation made by the variation in the intensity ratio. Moreover, it indicates that the 1D defects predominate the materials when 0D defects are reduced. Based on this finding, it is evident that carbon materials become more organized with a greater metal oxide loading.

Catalytic graphitization could occur through two different mechanisms: the dissolution–precipitation mechanism and the carbide formation–decomposition mechanism. Iron in the metal oxide product obtained from ilmenite belongs to the VIII group with six electrons in the d orbitals. They would scarcely accept electrons from carbon to form the carbide, and instead, iron would dissolve carbon to form positive ions. Hence, iron catalyzes the graphitization via the dissolution–precipitation mechanism. Titanium from ilmenite, which belongs to group IV, contains only two electrons in the d orbitals and could form strong bonds with carbon and hence produce carbide of titanium [55]. Therefore, titanium catalyzes graphitization via the carbide formation–decomposition mechanism. Hence, the metal oxide product obtained from ilmenite catalyzes the graphitization via both the dissolution–precipitation and carbide formation–decomposition mechanisms.

### 2.5. BET Analysis

**Figure 5:**
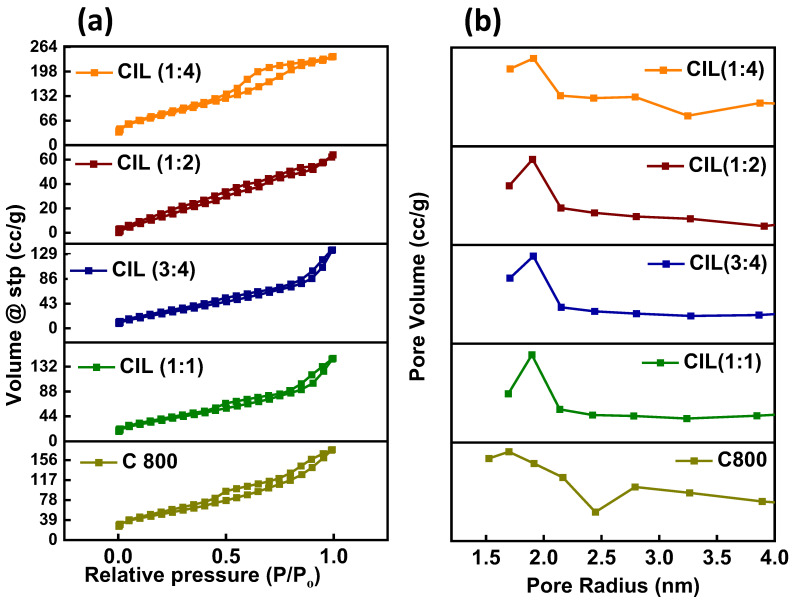
(**a**) Adsorption and desorption isotherms and (**b**) BJH pore size distribution curves of the synthesized materials.

Adsorption and desorption isotherms of the synthesized nanocomposites and the corresponding BJH pore size distribution curves are shown in Figure 5a,b, respectively. BET isotherms show the behavior of type V isotherms with H2 hysteresis loops, indicating that the synthesized materials consist of mesopores with narrow openings, which are relatively uniform channel-like pores. The surface areas of the pyrolyzed chitosan (C800) and the metal oxide product obtained from ilmenite (IL) were 77.57 and 301. 83 m^2^/g, respectively. The surface areas of all the CIL composites were greater than that of the carbonized chitosan because the composites consisted of a metal oxide product, which has a high surface area, in addition to the pyrolyzed chitosan. The surface area increased when the proportion of the ilmenite product rose from 50% (183.04 m^2^/g) to 57.14% (201.65 m^2^/g) due to the increased amount of high surface area metal oxide product incorporated. However, the surface area shrunk with further increments in the proportions 140.68 m^2^/g (66.67%) and 107.18 m^2^/g (75%), which was due to both clogging of the porous structure of the carbon structure and surface coverage of the metal oxide nanoparticles by the co-existing nanoparticles. The total pore volume of the composites decreased with an increasing proportion of the metal oxide product, in which the maximum total pore volume of 0.27 cc/g was obtained for CIL (1:1), and the minimum of 0.21 cc/g was evident for CIL (1:4).

The total pore volume of pyrolyzed chitosan was comparatively very low (0.10 cc/g), and the highest total pore volume (0.36 cc/g) was obtained for the ilmenite-based metal oxide product. The behavior of the pore radius in the pure materials and of the composites differed. The lowest pore radius of 2.4 nm was obtained for the ilmenite-based metal oxide product, and the pore radius of pyrolyzed chitosan was 2.5 nm. The highest pore radius among the composites (3.9 nm) was found with CIL (1:4), and the smallest (2.5 nm) was found with CIL (3:4). Interestingly, the lowest pore radius was reported in the composite with the highest surface area, while the highest pore radius was obtained in the composite with the smallest surface area.

### 2.6. XPS Analysis

**Figure 6:**
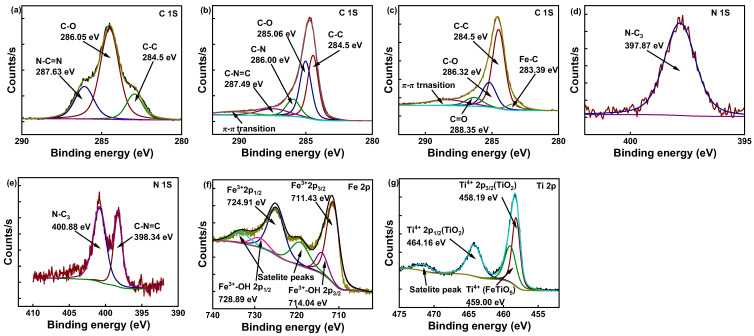
The higher resolution spectrum of (**a**) C 1s of chitosan, (**b**) C 1s of C 800, (**c**) C 1s of CIL (1:2), (**d**) N 1s of chitosan, (**e**) N 1s of C 800, (**f**) Fe 2p of IL and (**g**) Ti 2p of IL.

X-ray photoelectron spectra were collected to analyze the surface chemistry of the synthesized nanomaterials. The survey spectrum of pure chitosan and pyrolyzed chitosan (Supplementary Materials Figure S2a,b, respectively) showed the presence of carbon, oxygen and nitrogen. The higher resolution spectrum of C 1s of chitosan (Figure 6a) was deconvoluted into three peaks centered at 284.5, 286.05 and 287.63 eV, which are attributed to sp^2^ hybridized C-C, C-N and N-C=N, respectively [17]. The C 1s spectrum of pyrolyzed chitosan, C800 (Figure 6b), showed five subpeaks at 284.5, 285.06, 286, 287.49 and 290.51 eV, which were ascribed to sp^2^-hybridized C-C, C-O, C-N, N-C=N bonds and π–π interactions, respectively [17,56]. Similar behavior was observed in the higher-resolution spectra of C 1s of CIL (1:1) and CIL (3:4) composites (Supplementary Figure S2c,d, respectively). The higher resolution spectrum of C 1s of CIL (1:2) (Figure 6c) was deconvoluted into five peaks at 283.39, 284.5, 285.21, 286.32 and 288.35 eV, which are assigned to Fe-C in Fe_3_C, sp^2^-hybridized C-C, C-O, C=O, C-N and O-C=O, respectively [56,57,58]. The higher resolution spectrum of C 1s of CIL (1:4) (Supplementary Figure S2e) was deconvoluted into four peaks appearing at 283, 284.5, 285.87 and 287.96 eV, which can be attributed to the Fe-C in Fe_3_C, sp^2^-hybridized C-C, C-O and N-C=N, respectively. It is interesting to observe the occurrence of an Fe-C bond in Fe_3_C at the higher resolution spectrum of C 1s in CIL (1:2) and CIL (1:4). Indicated here is the formation of Fe_3_C with higher loading of the metal oxide product obtained from ilmenite.

The absence of peaks corresponding to Fe_3_C in the XRD patterns suggests the presence of Fe_3_C on the surface of the nanostructures. The higher resolution spectrum of N 1s of pure chitosan (Figure 6d) showed only one peak at 397.83 eV, which corresponds to a C-N bond of chitosan [59]. The higher resolution spectrum of N 1s of pyrolyzed chitosan (Figure 6e) was deconvoluted into two peaks at 398.34 and 400.8 eV, which correspond to C-N=C and N-C_3_, respectively [17]. The same chemical environment of nitrogen was observed in all four fabricated nanocomposites (CIL (1:1), CIL (3:4), CIL (1:2) and CIL (1:4)) as shown in Supplementary Figure S2f–i, respectively. The higher resolution spectrum of O 1s of C800 revealed in Supplementary Figure S2j shows the presence of oxygen bound to carbon via single and double bonds (C-O and C=O). The higher resolution spectra of O 1s of the nanocomposites CIL (1:1), CIL (3:4), CIL (1:2) and CIL (1:4) are shown in Supplementary Figure S2k–n, respectively. They were deconvoluted into three peaks at 529.85, 531.21 and 532.86 eV, which were assigned to the Ti^4+^/Fe^3+^- O, OH and C=O bonds, respectively [17].

The higher resolution spectrum of Fe 2p of the metal oxide product obtained from ilmenite, IL (Figure 6f), showed six peaks at 711.43, 714.04, 719.22, 724.91, 728.89 and 733.37 eV. They were assigned to Fe_2_O_3_, Fe-OH, satellite feature of 2p^3/2^, 2p^1/2^ of Fe^3+^, 2p^1/2^ of Fe^3+^ in Fe-OH and satellite feature of 2p^1/2^, respectively [60]. The higher resolution spectra of Fe 2p of the composites (Supplementary Figure S2o–r) consist of an additional peak at 710.4 eV, which represents the presence of 2p^3/2^ of Fe^3+^ of Fe_3_C. The higher resolution spectrum of Ti 2p of the metal oxide product obtained from the ilmenite, IL (Figure 6g), showed four peaks at 458.19, 459, 464.16 and 471.53 eV, which were assigned to the 2p^3/2^ Ti^4+^ of TiO_2_, 2p_3/2_ Ti^4+^ of Fe_2_TiO_5_, 2p^1/2^ of Ti^4+^ and to the satellite peak, respectively [61]. The higher resolution spectra of Ti 2p of the nanocomposites (Supplementary Figure S2s–v) showed only three peaks at 458.35, 464.05 and 471.4 eV, which were assigned to the 2p^3/2^, 2p^1/2^ Ti^4+^ of TiO_2_ and to the satellite feature, respectively.

### 2.7. FT-IR Analysis

**Figure 7:**
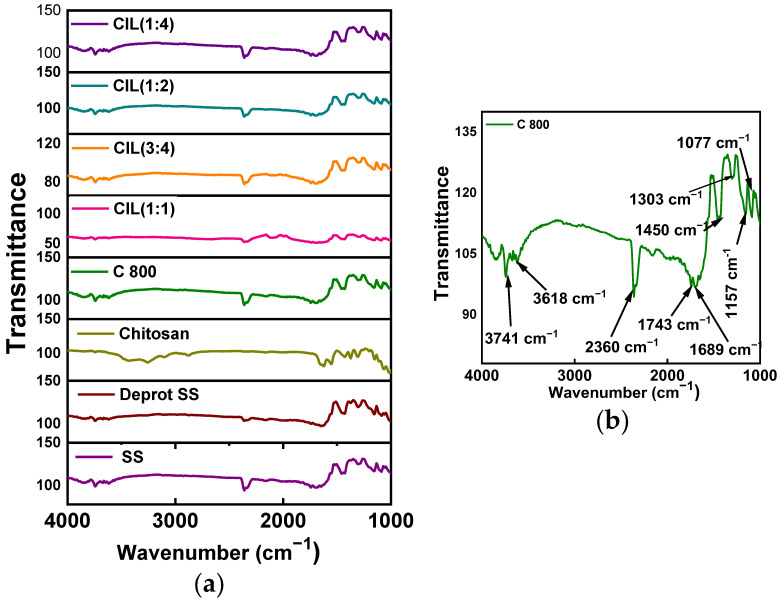
(**a**) FT-IR spectra of the synthesized materials and (**b**) FT-IR spectrum of chitosan.

FT-IR spectra were collected to determine the functional groups present on the synthesized materials (Figure 7a). The FT-IR spectrum for chitosan was given separately (Figure 7b) so that the vibrational bands could be better visualized. The vibrational band at 1087 cm^−1^ was attributed to the C-O stretching of primary and secondary alcohols, while the bands at 1157 and 1303 cm^−1^ were assigned to the C-O stretching in aliphatic ethers and C-O stretching in aromatic esters, respectively. The bands at 1458 and 2877 cm^−1^ were ascribed to the C-H bending and C-H stretching in alkane, respectively, while the band at 1697 cm^−1^ represents the C=O stretching in amides, which is present prominently in the FT-IR spectrum of chitin due to the presence of the -NHCOCH_3_ group. The presence of O-H stretching in carboxylic acids is indicated by the band at 3255 cm^−1^, and the bands at 3618 and 3741 cm^−1^ represent the O-H stretching in alcohols and in physiosorbed water. The band at 3433 cm^−1^ that was especially present in chitosan was attributed to the N-H stretching frequency of the -NH_2_ group in chitosan. Hence, the data obtained from the FT-IR and XPS analyses confirm the surface functional groups present in the synthesized nanocomposites.

### 2.8. DRS Analysis

**Figure 8:**
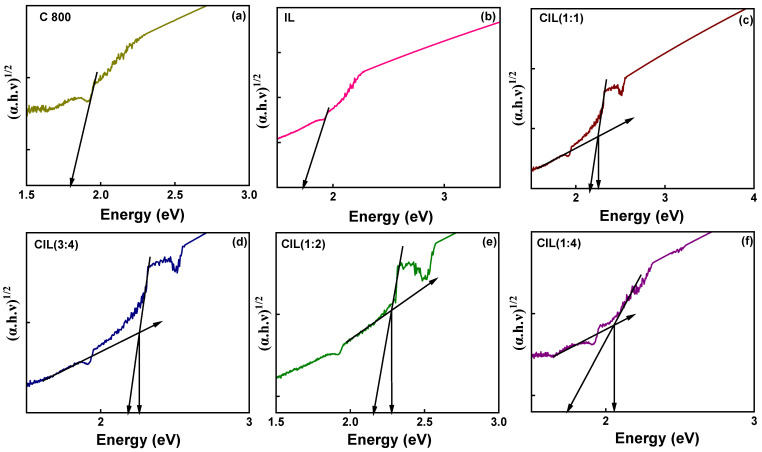
Tauc plots showing the indirect transitions of the synthesized materials. (**a**) C 800; (**b**) IL; (**c**) CIL(1:1); (**d**) CIL(3:4); (**e**) CIL(1:2); (**f**) CIL(1:4).

Diffuse reflectance spectra were acquired to study the absorption behavior of the synthesized composites. UV–visible spectra of the synthesized materials are shown in Supplementary Materials Figure S3. The UV–visible spectrum of the metal oxide product obtained from ilmenite (Supplementary Materials Figure S3a) reflected strong visible absorption with an absorption edge at about 544 nm, while that of nanocomposites exhibited similar higher visible absorption where the absorption edge lied at around 550 nm. Tauc plots ((F(R)* × hν)*n* vs. hν) were constructed for both direct and indirect transitions to determine the band gap of the synthesized nanomaterials, where *n* = 2 (Supplementary Materials Figure S3b) served to plot the direct transitions, and *n* = 1/2 constructed the Tauc plots corresponding to indirect transitions (Figure 8). Band gaps were calculated using the method proposed by Makula et al. [62]. The behaviors of the Tauc plots demonstrated that the materials showed the indirect transitions. The band gap value determined for the metal oxide product obtained from ilmenite was 1.78 eV, and the value for pyrolyzed chitosan was 1.80. The band gaps of CIL (1:1), CIL (3:4) and CIL (1:2) were about 2.26 eV, and the gap value of CIL (1:4) (2.06 eV) was slightly lower than the others. The band gap values calculated from direct and indirect transitions are tabulated in Table 2.

The crystalline metallic compounds and the crystalline carbon in the composites contribute to the band gap. The metallic content and hence the crystalline carbon content in CIL (1:4) was higher than that of the other composites, resulting in a smaller band gap compared to the others. Furthermore, as the crystalline metal and carbon are heterogeneously distributed in the material, the band gap would deviate without a particular trend when increasing the metal content. Moreover, the band gap depends on the particle size of the nanoparticles, where the band gap decreases with increasing particle size. The holes in the valence band and electrons in the conduction band confine with decreasing particle size; due to this confinement effect, the band gap increases. Furthermore, the shape of the nanomaterials also affects the band gap. The volume-to-surface ratio varies with the size and shape of the nanomaterial, which contributes to variation in the surface atoms and subsequently to the cohesive energy [63]. Therefore, the band gap varies at the nanoscale depending on the size and shape of the material. Hence, it is evident that the band gap of the nanomaterial depends on many factors and no clear trend was observed.

### 2.9. Photocatalytic Activity

**Figure 9:**
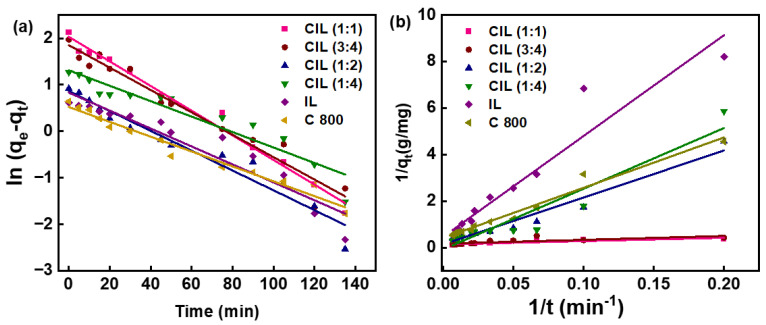
(**a**) Pseudo-first-order kinetics and (**b**) pseudo-second-order kinetics of adsorbing MB by the synthesized materials.

The photocatalytic activity of the synthesized nanocomposites was evaluated on the degradation of methylene blue under sunlight. Prior to this, the adsorption kinetics of the fabricated materials were determined by conducting the experiment in the dark. The adsorption kinetics were fitted to both the pseudo-first-order and pseudo-second-order kinetics models as shown in Figure 9a,b. As indicated by the correlation of coefficient (R^2^), the adsorption data of CIL (1:1) and CIL (3:4) were best fitted to pseudo-first-order kinetics, indicating the physisorption of MB to the composite surface. The adsorption data of CIL (1:2) and CIL (1:4) are best fitted to second-order kinetics, indicating chemisorption of methylene blue to the fabricated nanomaterials. The graphitization of carbon is caused by the metal oxides as described above in the Raman analysis, in which, upon graphitization, carbon becomes more ordered when atomic planes are arranged as shown in the HRTEM images. As the incorporated proportion of the metal oxide is high in the latter two, the degree of graphitization and hence the concentration of ordered carbon is high compared to CIL (1:1) and CIL (3:4). The planar MB molecules form π–π interactions, with the graphitic carbon facilitating the chemisorption.

**Figure 10:**
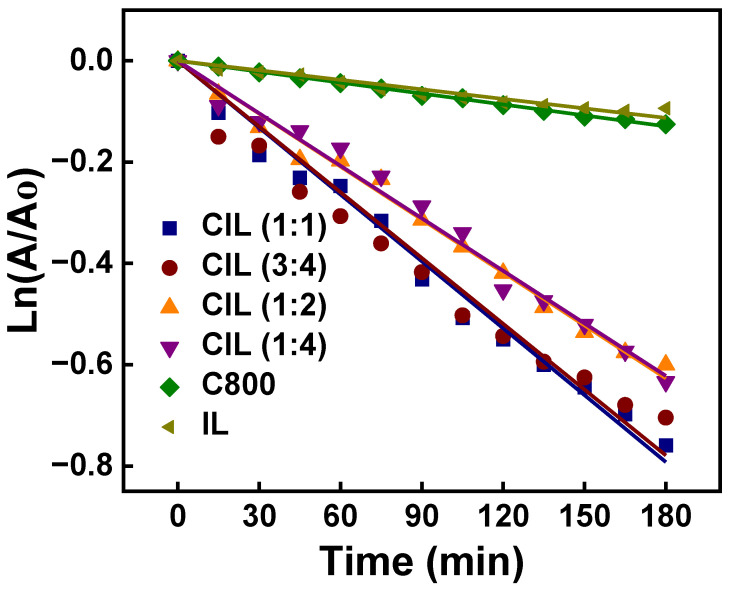
Pseudo-first-order kinetics of photocatalytic activity of the synthesized materials.

The synthesized photocatalysts were saturated with methylene blue prior to exposure to sunlight as described above. The highest rate constant for the degradation of methylene blue (4.4 × 10^−3^ min^−1^) was obtained with CIL (1:1) and CIL (3:4), while the rate constant for the degradation of MB in the presence of CIL (1:2) and CIL (1:4) was 3.5 × 10^−3^ min^−1^ (Figure 10). The rate constant per surface area was calculated, in which the highest rate constant/surface area (3.17 × 10^−5^ min^−1^) was obtained in the presence of CIL (1:4), while the lowest value (2.13 × 10^−5^ min^−1^) was obtained in the presence of CIL (3:4). All the rate constant values are tabulated in Table 3. The rate constants and the rate constant/surface area of both the pyrolyzed chitosan and metal oxide product obtained from ilmenite were lower than the nanocomposites.

**Figure 11:**
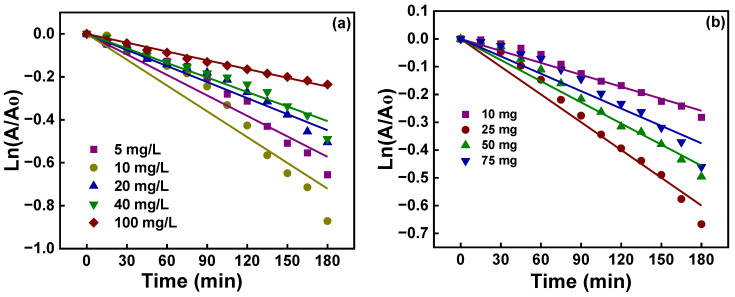
Effect of (**a**) initial dye concentration and (**b**) weight of catalyst on photocatalytic activity.

The overall reduction of MB by CIL (1:1) was larger than all other composites, and it was selected for further investigation. The effect of concentration of the MB dye on the photocatalytic activity was determined by varying the concentration to 5, 10, 20, 40 and 100 mg/L (Figure 11a). The highest rate constant (4.0 × 10^−3^ min^−1^) was obtained for the degradation of 10 mg/L MB, and the rate constant declined as MB concentration increased further. The rate constant for the degradation of 10 mg/L MB was 1.26 times greater than that for 5 mg/L MB, as the number of MB molecules is higher in 10 mg/L. However, with further increments in the dye concentration, the rate constant decreased because the available active sites in the catalyst increasingly did not sufficiently cater to the rising number of dye molecules (Table 4).

The effect of catalyst dosage on catalytic activity was evaluated by varying the weight of the catalyst as 10, 25, 50 and 100 mg (Figure 11b). It was interesting to see that the highest rate constant was obtained with 25 mg. The rate constant rose with an increasing catalyst dosage to 25 mg since the available number of active sites for the dye molecules to adsorb, and hence for the catalytic reaction, is high. However, with further increments in the weight of the catalyst, the rate of the reaction dropped because the solid catalyst particles covering the others restricted the mass transfer. This in turn limits the available surface for the reaction, leading to less catalytic activity (Table 5).

**Figure 12:**
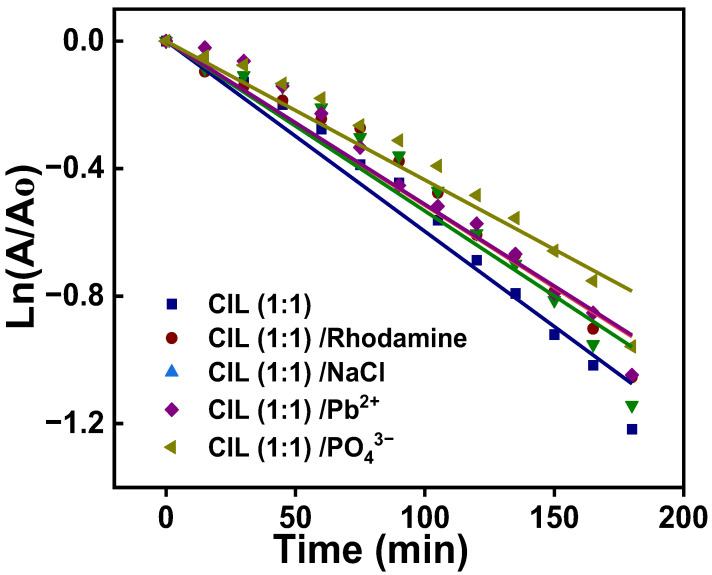
Effect of co-pollutants on photocatalytic activity.

Wastewater not only consists of dyes but is also abundant with other pollutants. The effect of co-pollutants on the photodegradation of methylene blue was determined by mixing MB with Pb^2+^ representing heavy metals, rhodamine B as another dye, NaCl as it changes the ionic strength and HPO_4_^2−^ representing fertilizers (Figure 12). It was observed that the rate constant for the photodegradation of MB abated in the presence of all co-pollutants, with a maximum reduction of 27% in the co-presence of Pb^2+^. Metal ions are positively charged small species that would easily interact electrostatically with the negatively charged catalyst surface limiting the active sites available for the dye molecules, which are also positively charged. Since the MB molecules are bulky and Pb^2+^ ions are small, due to the steric hindrance, adsorption of MB is limited compared to the Pb^2+^ ions, but rhodamine B is another positively charged dye molecule that has affinity to the catalyst surface similar to MB. The reduction in the rate of the photodegradation of MB was only 13%, and this demonstrates that degradation was not affected much by the presence of rhodamine B.

The reduction resulted from the co-adsorption of rhodamine B molecules to the active sites, and in the meantime, the rhodamine B dye molecules also get degraded by the generated radicals. Degradation of rhodamine B was also observed, and it degraded at a rate constant of 4.0 × 10^−3^ min^−1^, supporting the fact that rhodamine B was also co-degraded by radicals generated in the presence of the CIL (1:1) catalyst. The presence of NaCl also slowed down the rate of photodegradation of MB but only by 11% due to the clogging of active sites by Cl^−^ ions [64]. The rate of photodegradation was reduced by 14% due to the co-existence of the PO_4_^3−^, as they paired with the positively charged MB molecules; three MB molecules (+1 charged) couple with one phosphate ion (−3 charged), neutralizing the overall charge of MB and hence reducing the interaction of dye molecules with the catalyst surface [65]. The variations in the rate constant in the presence of co-pollutants on the photodegradation of MB are stated in Table 6.

**Figure 13:**
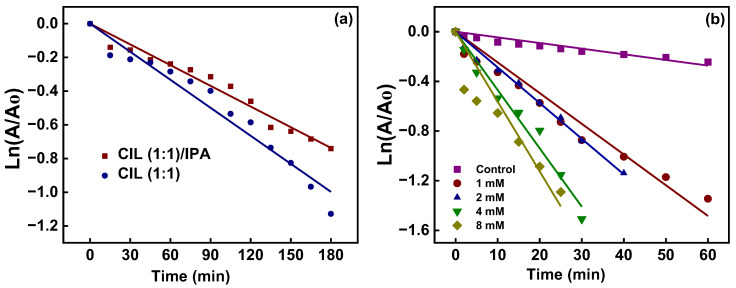
(**a**) Effect of IPA and (**b**) effect of persulfate concentration on photocatalytic activity.

The exact reactive species that are responsible for the photodegradation of MB were determined by conducting the photocatalytic experiment in the presence of isopropyl alcohol and EDTA as the holes and hydroxyl radical scavengers, respectively. It was noticed that the rate of reaction declined from 5.5 × 10^−3^ min^−1^ to 4.1 × 10^−3^ min^−1^, which suggested that the hydroxyl radicals are responsible for the degradation of MB (Figure 13a). The effect of EDTA was also monitored. Interestingly, it was observed that the blue color suddenly disappeared upon exposure to sunlight, but with gentle shaking, the blue color reappeared. This observation was reproducible, and it is believed that EDTA facilitates the rapid physisorption of MB to the catalyst surface [66,67].

The effect of persulfate ions on the degradation of MB was studied varying the concentration of persulfate ions as 1, 2, 4 and 8 mM (Figure 13b). It was noted that the rate constant dramatically increased in the presence of persulfate ions in general, and the rate constant increased 5.4-, 6.3-, 10.3- and 12.4-fold, respectively, utilizing the abovementioned concentrations. Table 7 shows the increase in the rate constants with increasing concentrations of persulfate.

Persulfate ions produce both SO_4_^−●^ and OH^●^ by the reduction of S_2_O_8_^2−^ in the presence of photons. S_2_O_8_^2−^ ions are reduced to SO_4_^−●^, as shown in reaction 1, by photogenerated electrons, and the produced SO_4_^−●^ reacts with OH- in the medium to produce SO_4_^2−^ and OH^●^ (reaction 2). Furthermore, in the presence of UV light, S_2_O_8_^2−^ degrades to produce 2SO_4_^−●^ (reaction 3), which would react with OH^−^ to produce OH^●^. Moreover, in the presence of O_2_^−●^, S_2_O_8_^2−^ produces SO_4_^2−^, SO_4_^−●^ and O_2_, as shown in reaction 4.

S_2_O_8_^2−^ + e^−^ → SO_4_^−●^ + SO_4_^2−^ (5)

SO_4_^−●^ + OH^−^ → SO_4_^2−^ + OH^●^ (6)

S_2_O_8_^2−^ + hν → 2 SO_4_^−●^ (7)

S_2_O_8_^2−^ + O_2_^−●^ → SO_4_^2−^ + SO_4_^−●^ + O_2_ (8)

### 2.10. Photocatalytic Mechanism

**Figure 14:**
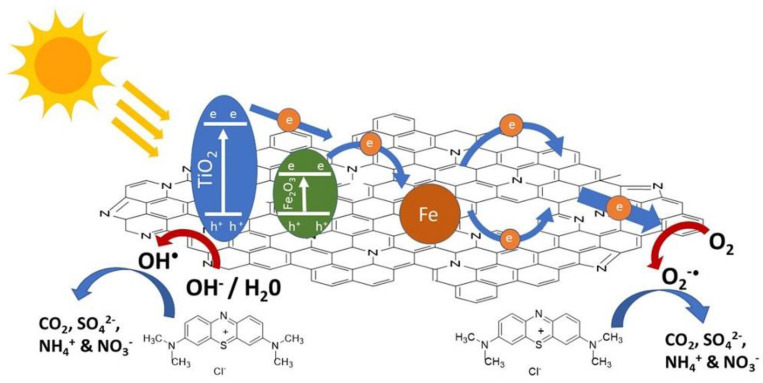
Mechanism of photocatalysis.

TiO_2_ semiconductors supported on the graphitic carbon matrix are excited due to the UV component of sunlight, while Fe_2_O_3_ is excited by visible light. The excited electrons at the CB of the semiconductors are transferred to the conductive graphitic carbon matrix, and such electrons are further captured by metallic Fe. The electrons are then eventually transferred to molecular O_2_ to produce O_2_^−●^. H_2_O oxidized to OH^●^ and the released electrons were attracted by the holes generated at the VB of TiO_2_ and Fe_2_O_3_. Both O_2_^−●^ and OH^●^ radicals oxidized MB to harmless products. The proposed mechanism is shown in Figure 14.

### 2.11. Reusability

**Figure 15:**
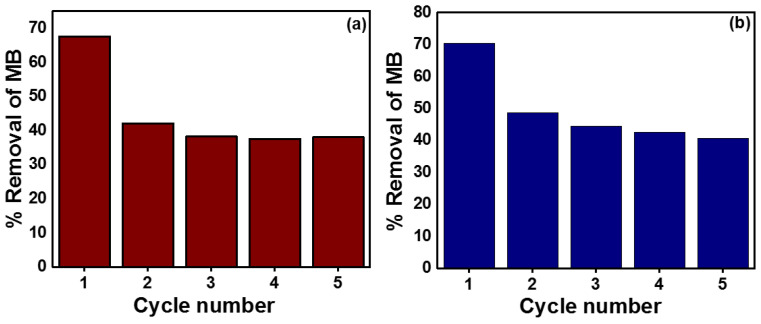
Removal of MB in five consecutive cycles (**a**) with washing and (**b**) without washing the catalyst.

The reusability of the CIL (1:1) was examined in two different ways. In one way, the catalyst was washed before using it again for the next time, and in the other way, it was also used directly without washing for the consecutive cycle (Figure 15a,b, respectively). This was to establish the commercial applicability of the catalyst because washing the catalyst may not be favorable since washing consumes the solvent, time and energy. In both circumstances, it was observed that the catalytic conversion was reduced moving from one cycle to the other, where a reduction of 43% was observed at the fifth cycle, suggesting that the net conversion of MB to harmless products by photocatalysis is not influenced by washing the catalyst. Hence, the use of CIL (1:1) is much more commercially viable. However, reduction in the conversion moving from one cycle to another was caused by: (1) blocking of the active sites from the chemisorbed molecules; (2) reduction in the surface area due to the agglomeration of the solid catalyst particles and (3) the washout of the catalyst metal-based nanoparticles from the graphitic carbon matrix. The reduction in the conversion of MB moving from the first cycle to the second is significantly greater compared to the reduction between other consecutive cycles. This could have resulted mainly due to the washout of the metal-based nanoparticles, which further suggests that many metal-based nanoparticles are located on the surface of the graphitic carbon matrix instead of being embedded in the framework. Therefore, loosely bound metal-based nanoparticles wash out greatly moving from the first cycle to the second during the catalytic reaction and/or washing step, which led to a significant loss in photocatalytic activity.

It is evident that the fabricated nanocomposite is effective in degrading MB; it is superior to some composites, while its performance was lower than some other photocatalysts (Table 8). However, it should be noted that the rate constant for the degradation of MB depends on the dye concentration, dosage of the catalyst, the light source, power, lux value etc. However, it is worth emphasizing that the photocatalyst reported in this study not only degraded dyes to help environmental remediation, but it also added value to a waste product (shrimp shells) and natural mineral (ilmenite).

## 3. Materials and Methodology

### 3.1. Chemicals and Materials

*Penaeus vannamei* (shrimp) shells were sourced from Ceylon Catch (Pvt) Ltd., while ilmenite sand was supplied by Lanka Minerals Sand (Pvt) Ltd., Sri Lanka. HCl (37%) and HNO_3_ (68%) were procured from Sigma Aldrich (Gillingham SP8 4XT, United Kingdom). NaOH pellets were purchased from Sisco Research Laboratories (Pvt) Ltd. (Mumbai, India), and methylene blue was supplied by Himedia Leading Biosciences Company (Maharashtra, India). Nano-pure water was used for all the experiments. All the chemicals utilized in the experiments were of analytical grade and utilized without further purification.

### 3.2. Dissolution of Ilmenite Sand and Precipitation

An amount of 3 g of ilmenite sand was treated with 200 mL of conc. HCl under refluxing conditions at 90 °C for 5 h. The acid-digested ilmenite product was separated, and the residue was again treated with a new 200 mL of HCl. The same procedure was repeated four times. All five leachates obtained were combined, and conc. NH_4_OH was added dropwise until the pH reached 10. Then the obtained brown color precipitate was washed with nano-pure water until the Cl^−^ ions were removed and neutral pH was reached. The resulting precipitate was dried at 80 °C. This product will be abbreviated as IL in the text.

### 3.3. Synthesis of Chitosan from Shrimp Shells

The sourced shrimp shells were washed several times with nano-pure water followed by two rounds of washing with isopropyl alcohol to remove impurities. The washed shells were then dried in an oven at 60 °C and crushed using a grinder until a fine powder consistency was achieved. Demineralization/removal of CaCO_3_ and Ca_3_(PO_4_)_2_ from shrimp shell powder was carried out next. The shell powder was suspended in a 10% (*v*/*v*) HCl solution for 24 h. Then, the acid was washed off with nano-pure water until neutral pH was achieved. The powder was then left in a 3% NaOH solution for 5 h while stirring to remove the proteins. Deacetylation was done next by refluxing the powder in 50% NaOH for 2 h at 80 °C, where chitin from the shells was converted to chitosan. After that, the powder was thoroughly washed with nano-pure water to remove the base, and a neutral pH was obtained. Chitosan was then dried in an oven stored in an airtight container for further use and analysis. The product obtained will be referred to as C800 in the text.

### 3.4. Fabrication of the Nanocomposite

First, chitosan was mixed with 2 mM HNO_3_ acid and sonicated for 10 min. Then, the metal oxide product obtained from ilmenite (IL) was added to it in the proper weight ratio and sonicated for another 30 min. The sample was hydrothermally treated in a 2 mM HNO_3_ acid solution at 180 °C for 15 h. The hydrothermally treated sample was filtered and washed with distilled water until a neutral pH was achieved. The mixture was annealed at 800 °C for 2 h in a N_2_ environment. Samples were synthesized in different ratios of chitosan and IL as follows: IL:chitosan, 1:1, 1:0.75, 1:0.5 and 1:0.25. These are abbreviated in the text, respectively, as CIL (1:1), CIL (3:4), CIL (1:2) and CIL (1:4).

### 3.5. Photocatalytic Activity

The photocatalytic activity of the synthesized nanocomposites was evaluated on the degradation of the methylene blue dye under sunlight. In a typical experiment, 25 mg of the nanocomposites was shaken in 25 mL of a 25 mg/L methylene blue aqueous solution for 18 h to reach the adsorption–desorption equilibrium. Then the solution was decanted and resuspended in 50 mL of 10 mg/L methylene blue solution and kept in the dark for 30 min. The samples were subsequently exposed to sunlight, and during that period, aliquots were withdrawn at 15-min intervals, and the samples were analyzed using a UV–Visible spectrophotometer. Experiments were conducted between 11.00 a.m. and 3.00 p.m. in September 2022, where the lux value varied in the 80,000–1,200,000 lux range during the experiments.

## 4. Characterization

The D8 Advance Bruker system, which uses Cu Kα (λ = 0.154 nm) radiation varying the 2θ from 5° to 80° at a scan speed of 2°/min, was utilized to obtain XRD patterns. A transmission electron microscope (JEOL─JEM-2100) operated with 200 kV served to characterize the morphology of the synthesized nanocomposites. A volume of 1 μL of nanomaterial dispersed in ethanol was mounted on a carbon copper grid with holes and allowed to dry at room temperature prior to TEM analysis. The energy-dispersive X-ray (EDS) spectra were collected with the EDAX element EDS system, while scanning electron microscopic (SEM) images were acquired using a Carl ZEISS EVO 18 RESEARCH instrument. The chemical compositions of the samples were studied by X-ray fluorescence (XRF) using a HORIBA Scientific XGT-5200 X-ray analytical microscope, which was possessed with a Rh anode X-ray tube operated at a maximum voltage of 50 kV. The Thermo Scientific ESCALAB Xi+ X-ray photoelectron spectrometer functioned to acquire the survey spectra and higher-resolution spectra of the synthesized catalysts. To collect the diffuse reflectance spectra of the prepared samples, the Shimadzu 1800 UV–visible spectrophotometer, which uses a precision Czerny–Turner optical system, was utilized. Measurements were obtained through the 400 nm to 750 nm range with a bandwidth of 1.0 nm where the wavelength accuracy was ±0.1 nm. A Bruker Senterra Raman microscope spectrophotometer was used to carry out the Raman analysis. The Carbolite Gero tube furnace annealed the samples, and the absorbance of methylene blue samples was measured using a Shimadzu UV-1990 double-beam UV–visible spectrophotometer.

## 5. Conclusions

In conclusion, we fabricated TiO_2_-Fe_2_O_3_-Fe/nitrogen-doped graphitic carbon composites using waste product shrimp shells and natural ilmenite sand as raw materials. All the composites effectively removed methylene blue by adsorption. CIL (1:1) showed the highest adsorption capacity (8.36 mg/g), removing 23% of MB molecules, which were adsorbed to the nitrogen-doped graphitic carbon via π–π interactions. The fabricated nanocomposites were effective at degrading MB under sunlight. The highest rate constant (4.4 × 10^−3^ min^−1^) for the degradation of MB was achieved with both CIL (1:1) and CIL (3:4), in which overall maximum MB removal was possible with CIL (1:1). The photocatalytic activity depended on the dye concentration and weight of the catalyst, where the maximum activity was accomplished with 10 mg/L and 25 mg, respectively.

Minimal photocatalytic activities were obtained at higher dye concentrations, in which the minimum of 1.4 × 10^−3^ min^−1^ was found when 100 mg/L of MB was used. The rate constant generally decreased with increasing weight of the catalyst, where the minimum of 2.1 × 10^−3^ min^−1^ was obtained with 100 mg of the catalyst. This resulted due to the limited number of active sites to cater to the large number of dye molecules and the limitation of mass transfer, respectively. Hydroxyl radicals were mainly responsible for the degradation of MB, and photocatalytic activity was enhanced by persulfate ions due to the production of both SO_4_^−●^ and OH^●^. The activity increased with increasing S_2_O_8_^2−^ concentration, in which the maximum rate constant of 5.6 × 10^−2^ min^−1^ was obtained when 8 mM of S_2_O_8_^2−^ was used. Photogenerated electrons by TiO_2_, Fe_2_O_3_ and Fe species were migrated through the conductive nitrogen-doped graphitic carbon matrix and eventually reduced molecular O_2_ to produce O_2_^−●^. The holes oxidized H_2_O and OH^−●^, where both O_2_^−●^ and OH^−●^ oxidized MB molecules to harmless products. Thus, nanocomposites fabricated by natural ilmenite sand and waste product shrimp shells are very employable for environmental remediation projects. The effect of the fabricated photocatalysts on degradation of different types of dyes and on different organic pollutants such as pesticides and pharmaceuticals could be determined in the future.

## Figures and Tables

**Table 1:** Crystallographic parameters calculated from XRD data.

Sample	Plane	2θ (°)	L (nm)	d (nm)	L/d
CIL (1:1)	(104)	32.82	39.83	0.2726	146
CIL (3:4)	(104)	32.82	39.29	0.2727	144
CIL (1:2)	(104)	32.89	39.84	0.2721	146
CIL (1:4)	(104)	32.74	55.39	0.2733	202

**Table 2:** The band gap values calculated from direct and indirect transitions.

Sample	Band Gap (eV)	
	Direct (*n* = 2)	Indirect (*n* = 1/2)
CIL (1:1)	2.28	2.26
CIL (3:4)	2.28	2.26
CIL (1:2)	2.18	2.28
CIL (1:4)	2.31	2.06
C800	1.84	1.80
IL	1.87	1.78

**Table 3:** Pseudo-first-order rate constants for the photodegradation of MB.

Sample	Pseudo-First-Order Kinetics		
	R^2^	Rate Constant (min^−1^) × 10^−3^	Rate Constant/Surface Area × 10^−5^
CIL (1:1)	0.9954	4.4	2.4
CIL (3:4)	0.9892	4.3	2.1
CIL (1:2)	0.9973	3.5	2.5
CIL (1:4)	0.9957	3.4	3.2
IL	0.9853	0.63	0.21
C800	0.9990	0.72	0.93

**Table 4:** Pseudo-first-order rate constants showing the effect of dye concentration on the photocatalytic activity.

Concentration (mg/L)	Pseudo-First-Order	
	R^2^	Rate Constant × 10^−3^ (min^−1^)
5	0.9667	3.2
10	0.9611	4.0
20	0.9861	2.5
40	0.9832	2.2
100	0.9970	1.4

**Table 5:** Pseudo-first-order rate constants showing the effect of weight of the catalyst on the photocatalytic activity.

Mass (mg)	Pseudo-First-Order	
	R^2^	Rate Constant × 10^−3^ (min^−1^)
10	0.9862	1.4
25	0.9890	3.3
50	0.9893	2.5
100	0.9679	2.1

**Table 6:** Pseudo-first-order rate constants showing the effect of co-pollutants on the photocatalytic activity.

Co-Pollutant	Pseudo-First-Order	
	R^2^	Rate Constant × 10^−3^ (min^−1^)
Control	0.9889	6.0
Rhodamine	0.9859	5.1
NaCl	0.9752	5.3
Phosphate	0.9867	5.1
Lead nitrate	0.9762	4.4

**Table 7:** Variations in the pseudo-first-order rate constant by increasing the concentration of persulfate ions.

Concentration	R^2^	Rate Constant (min^−1^)
Control	0.9670	4.55 × 10^−3^
1 mM	0.9823	2.47 × 10^−2^
2 mM	0.9954	2.83 × 10^−2^
4 mM	0.9889	4.69 × 10^−2^
8 mM	0.9422	5.62 × 10^−2^

**Table 8:** A comparison of the rate constant respective to the other parameters in different studies.

Photocatalysts	Dosage (g/L)	MB Concentration (mg/L)	Rate (min^−1^)	Light Source	Reference
TiO_2_/Fe_2_O_3_/Fe-/N-doped Graphitic Carbon Composite	0.5	10	4.4 × 10^−3^	Natural sunlight	Present study
TiO_2_/Seashell Composites	1	10	1.76 × 10^−2^	UV light (365 nm)	[38]
TiO_2_-Fe_3_C-Fe-Fe_3_O_4_/Graphitic Carbon Composites	1	10	7.0 × 10^−3^	Natural sunlight	[58]
Fe_2_TiO_5_/TiO_2_	2	10	8.4 × 10^−3^	Natural sunlight	[68]
TiO_2_ Nanoparticles Sensitized with Natural Chlorophyll Pigments	2.5	20	1.4 × 10^−2^	Visible blue LED light (457 nm)	[69]

## Data Availability

Data is contained within the article or supplementary material.

## Supplementary Materials

The following supporting information can be downloaded at: https://www.mdpi.com/article/10.3390/molecules28073154/s1, Figure S1: Enlarged versions of the XRD patterns showing the absence of the (104) peak of calcite; Figure S2: The survey spectrum of (a) pure chitosan (b) pyrolyzed chitosan, the higher resolution spectra of C 1s of (c) CIL (1:1) (d) CIL (3:4) ^®^ CIL (1:4), the higher resolution spectra of N 1s of (f) CIL (1:1), (g) CIL (3:4), (h) CIL (1:2) (i) CIL (1:4), the higher resolution spectra of O 1s of (j) pure chitosan (k) CIL (1:1), (l) CIL (3:4), (m) CIL (1:2) (n) CIL (1:4), the higher resolution spectra of Fe 2p of (o) CIL (1:1), (p) CIL (3:4), (q) CIL (1:2) ^®^ CIL (1:4), the higher resolution spectra of Ti 2p (s) CIL (1:1), (t) CIL (3:4), (u) CIL (1:2) (v) CIL (1:4); Figure S3: (a) Absorption vs. wavelength (b) Tauc plots showing the indirect transitions of the synthesized materials.
