# Supplementary material for: Fabrication of Naturally Derived Chitosan and Ilmenite Sand-Based TiO2/Fe2O3/Fe-N-Doped Graphitic Carbon Composite for Photocatalytic Degradation of Methylene Blue under Sunlight
Source: Molecules. 2023 Apr 1;28(7):3154. doi: 10.3390/molecules28073154 (PMC10096480; doi:10.3390/molecules28073154)
Supplement: Supplementary file 1 [file molecules-28-03154-s001.zip › molecules-2316140-supplementary.pdf]

Supplementary Information

Fabrication of Naturally Derived Chitosan and Ilmenite Sand-Based TiO_2/Fe_2O_3/Fe-N-Doped Graphitic Carbon Composite for Photocatalytic Degradation of Methylene Blue under Sunlight

Amavin Mendis [1], Charitha Thambiliyagodage [1,*], Geethma Ekanayake [1], Heshan Liyanaarachchi [1], Madara Jayanetti [1] and Saravanamuthu Vigneswaran [2,3,*]

[1] Faculty of Humanities and Sciences, Sri Lanka Institute of Information Technology, Malabe 10115, Sri Lanka
[2] Faculty of Engineering and Information Technology, University of Technology Sydney, P.O. Box 123, Sydney, NSW 2007, Australia
[3] Faculty of Sciences & Technology (RealTek), Norwegian University of Life Sciences, P.O. Box N-1432 Ås, Norway
* Correspondence: charitha.t@sliit.lk (C.T.); saravanamuth.vigneswaran@uts.edu.au (S.V.)

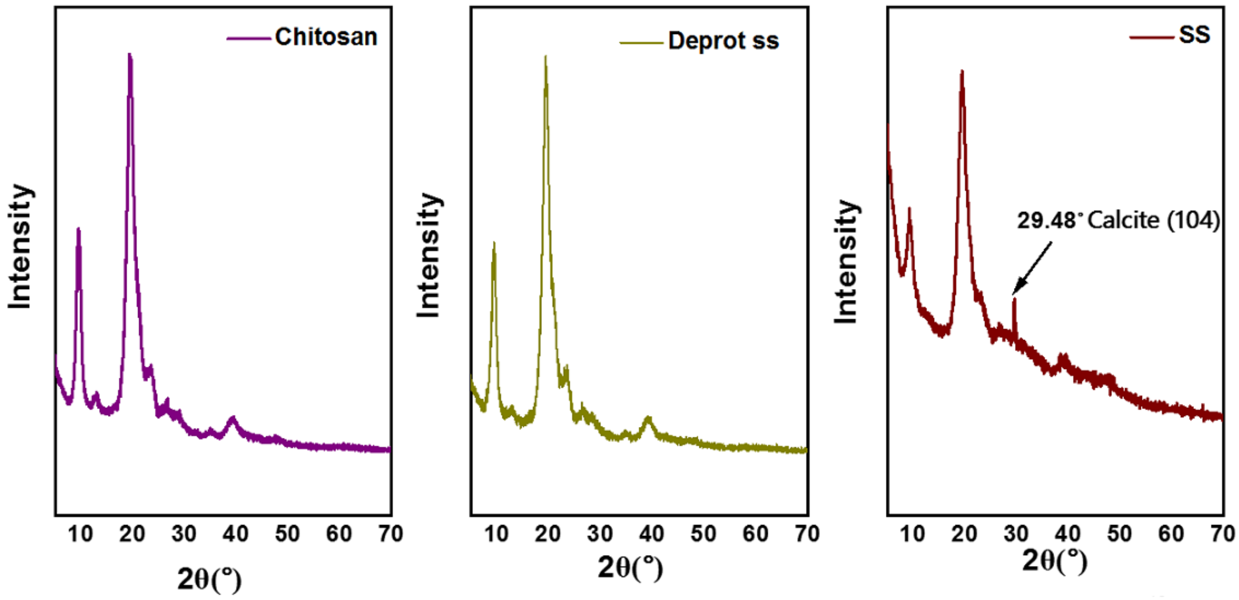

Figure S1. Enlarged versions of the XRD patterns showing the absence of the (104) peak of calcite.

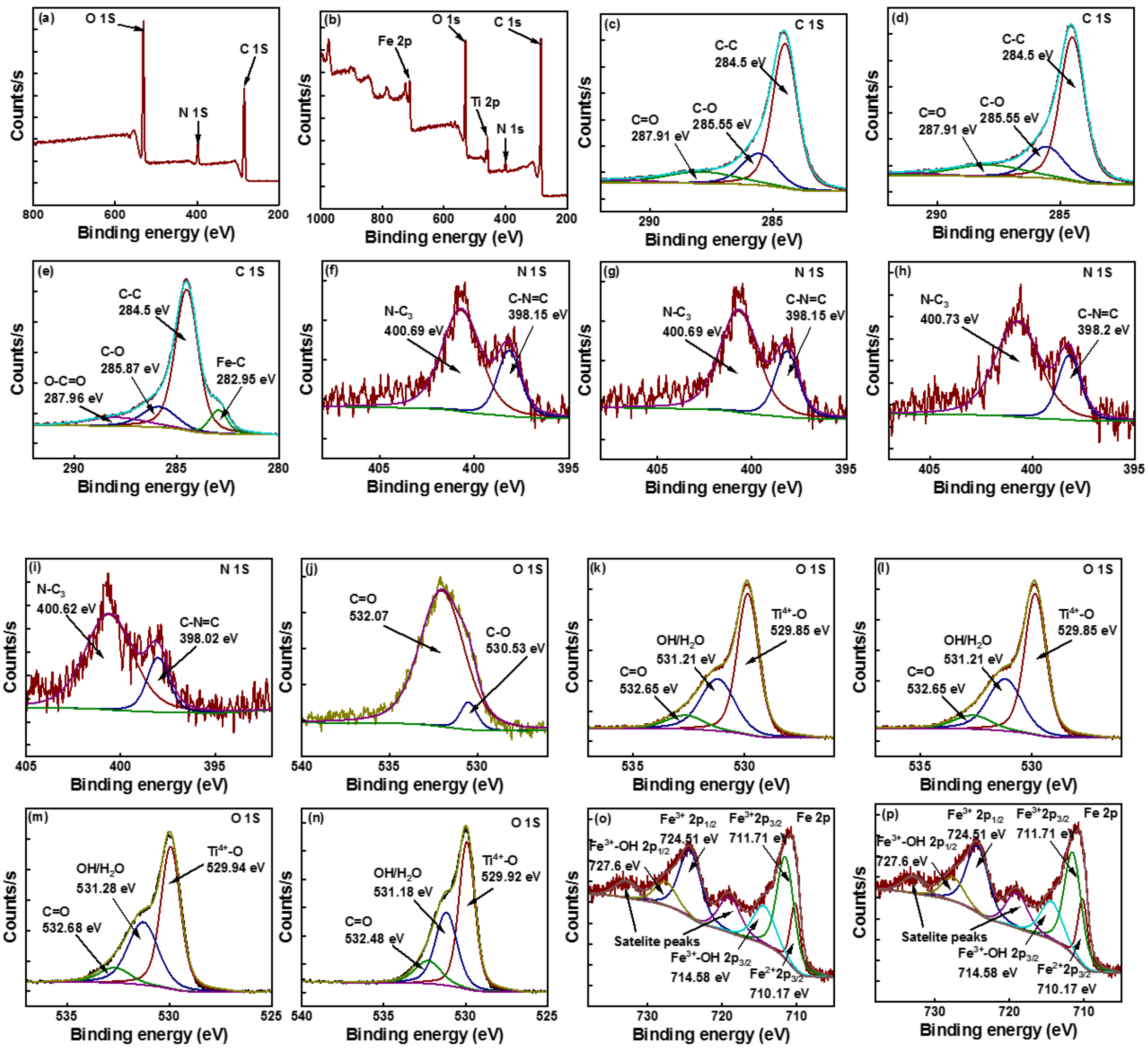
(a)
O 1S
N 1S
C 1S
Counts/s
Binding energy (eV)
(b)
Fe 2p
O 1s
C 1s
Ti 2p
N 1s
(c)
C 1S
C-C
284.5 eV
C-O
285.55 eV
C=O
287.91 eV
(d)
C 1S
C-C
284.5 eV
C-O
285.55 eV
C=O
287.91 eV
(e)
C 1S
C-C
284.5 eV
C-O
285.87 eV
O-C=O
287.96 eV
Fe-C
282.95 eV
(f)
N 1S
N-C3
400.69 eV
C-N=C
398.15 eV
(g)
N 1S
N-C3
400.69 eV
C-N=C
398.15 eV
(h)
N 1S
N-C3
400.73 eV
C-N=C
398.2 eV
(i)
N 1S
N-C3
400.62 eV
C-N=C
398.02 eV
(j)
O 1S
C=O
532.07
C-O
530.53 eV
(k)
O 1S
OH/H2O
531.21 eV
C=O
532.65 eV
Ti4+-O
529.85 eV
(l)
O 1S
OH/H2O
531.21 eV
C=O
532.65 eV
Ti4+-O
529.85 eV
(m)
O 1S
OH/H2O
531.28 eV
C=O
532.68 eV
Ti4+-O
529.94 eV
(n)
O 1S
OH/H2O
531.18 eV
C=O
532.48 eV
Ti4+-O
529.92 eV
(o)
Fe 2p
Fe3+ 2p1/2
724.51 eV
Fe3+2p3/2
711.71 eV
Fe3+-OH 2p1/2
727.6 eV
Satelite peaks
Fe3+-OH 2p3/2
714.58 eV
Fe2+2p3/2
710.17 eV
(p)
Fe 2p
Fe3+ 2p1/2
724.51 eV
Fe3+2p3/2
711.71 eV
Fe3+-OH 2p1/2
727.6 eV
Satelite peaks
Fe3+-OH 2p3/2
714.58 eV
Fe2+2p3/2
710.17 eV

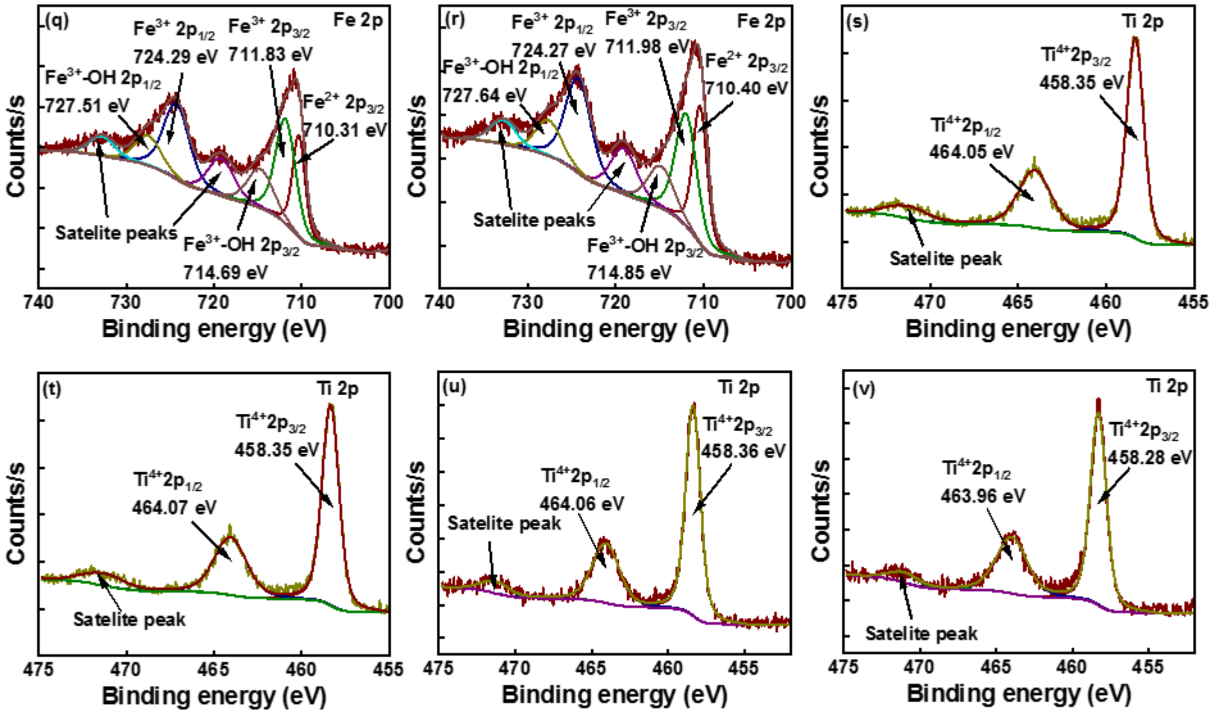

Figure S2. The survey spectrum of (a) pure chitosan (b) pyrolyzed chitosan, the higher resolution spectra of C 1s of (c) CIL (1:1) (d) CIL (3:4) (e) CIL (1:4), the higher resolution spectra of N 1s of (f) CIL (1:1), (g) CIL (3:4), (h) CIL (1:2) (i) CIL (1:4), the higher resolution spectra of O 1s of (j) pure chitosan (k) CIL (1:1), (l) CIL (3:4), (m) CIL (1:2) (n) CIL (1:4), the higher resolution spectra of Fe 2p of (o) CIL (1:1), (p) CIL (3:4), (q) CIL (1:2) (r) CIL (1:4), the higher resolution spectra of Ti 2p (s) CIL (1:1), (t) CIL (3:4), (u) CIL (1:2) (v) CIL (1:4).

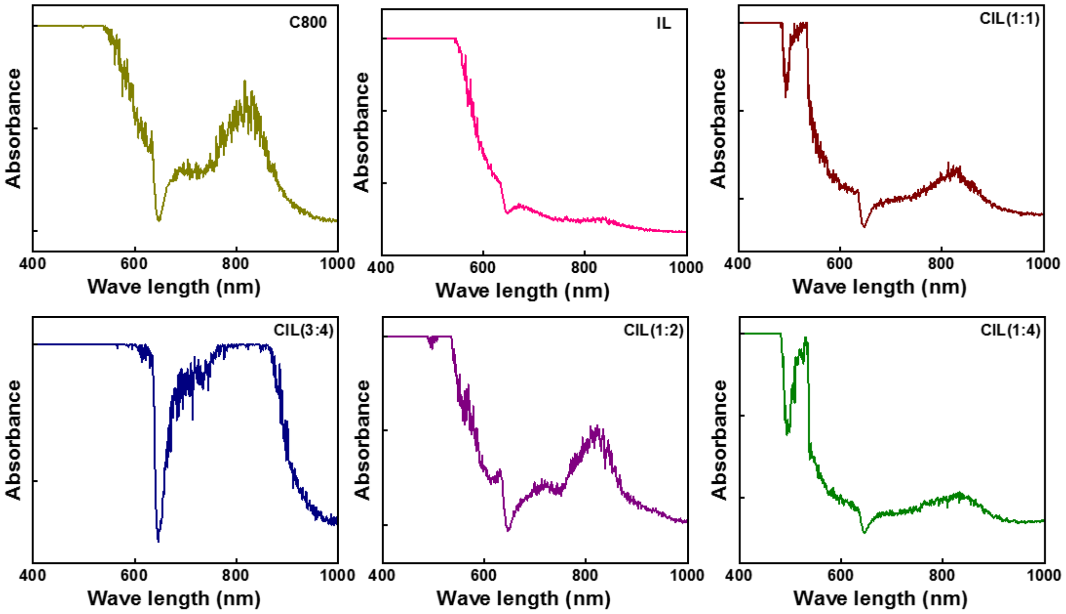

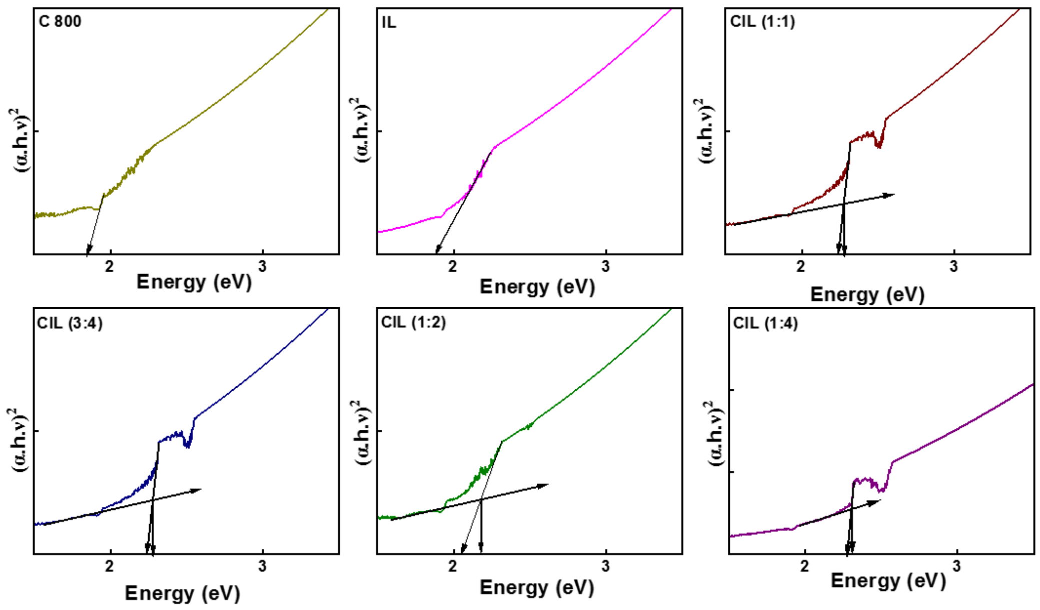

Figure S3. (a) Absorption vs wavelength (b) Tauc plots showing the indirect transitions of the synthesized materials.
